# Adaptation of potato cultivars to phosphorus variability and enhancement of phosphorus efficiency by *Bacillus subtilis*

**DOI:** 10.1186/s12870-024-05868-x

**Published:** 2024-12-12

**Authors:** Leangsrun Chea, Mohammad Alhussein, Petr Karlovsky, Elke Pawelzik, Marcel Naumann

**Affiliations:** 1 Quality of Plant Products, Department of Crop Sciences, University of Goettingen, Carl-Sprengel-Weg 1, 37075 Goettingen, Germany; 2 Molecular Phytopathology and Mycotoxin Research, Department of Crop Sciences, University of Goettingen, Grisebachstraße 6, 37077 Goettingen, Germany; 3 Present address: Center of Excellence on Sustainable Agricultural Intensification and Nutrition, Royal University of Agriculture, Dangkor District, Phnom Penh, Cambodia; 4 Present address: Plant Nutrition and Crop Physiology, University of Goettingen, Carl-Sprengel-Weg 1, 37075 Goettingen, Germany

**Keywords:** Plant growth-promoting *Bacillus subtilis*, Potatoes, Phosphorus, Phosphorus use efficiency, Root system

## Abstract

**Background:**

Plants utilize a variety of mechanisms to adapt to fluctuations in phosphorus (P) availability. Potatoes, in comparison to other crops, often display reduced phosphorus use efficiency (PUE) due to their underdeveloped root systems; therefore, understanding the mechanisms underlying PUE is critical for improving it. This study aimed to evaluate the morphological and physiological responses of potatoes to different P levels, with a focus on root system alterations and PUE. Two potato cultivars, a table potato (cv. Milva) and a starch potato (cv. Lady Claire), were subjected to varying P levels (0.5, 2, 5, and 30 mg P L^-1^ supplied as KH_2_PO_4_) in a hydroponic system. Additionally, the plants grown under 0.5 and 2 mg P L^-1^ were treated with plant growth-promoting *Bacillus subtilis* (*B. subtilis*), compared to untreated controls, to investigate the effectiveness of *B. subtilis* in addressing P deficiency. *B. subtilis* inoculation was performed by adding a bacterial suspension weekly to the hydroponic nutrient solution.

**Results:**

The findings illustrated Milva’s ability to efficiently allocate P and sugars to its roots under low P levels, thereby enhancing biomass and facilitating increased P uptake and PUE. Conversely, Lady Claire exhibited lower P assimilation efficiency under low P levels but demonstrated improved efficiency under high P availability. The concentration of P in the nutrient solution affected P uptake and several factors believed to be involved in P utilization, such as root morphology, sugar and indole-3-acetic acid concentration in the roots, and acid phosphatase activity. Gene expression analyses underscored the pivotal roles of *StPHT1;1* and *StPHT2;1* in P translocation to shoots, particularly in Lady Claire. Inoculation with *B. subtilis* improved P acquisition efficiency by 10% under low phosphorus levels (P0.5 and P2), particularly in Lady Claire, where shoot and root phosphorus contents increased by 13–25% and 4–13%, respectively. Additionally, *B. subtilis* displayed higher efficacy in mitigating P deficiency in Lady Claire compared to Milva, particularly under low P levels (P0.5 and P2).

**Conclusion:**

Milva showed greater phosphorus efficiency than Lady Claire under low P conditions, attributed to higher P and sugar levels in roots, enhancing root growth, P uptake, and translocation to shoots, particularly to young leaves. However, Lady Claire demonstrated a notable increase in P uptake and enhanced responsiveness to *B. subtilis* inoculation, particularly under low P levels (P0.5 and P2). These findings provide valuable insights for optimizing P management strategies to improve PUE in potatoes, especially under low P levels.

**Supplementary Information:**

The online version contains supplementary material available at 10.1186/s12870-024-05868-x.

## Introduction

Phosphorus (P) stands as a pivotal nutrient for plant vitality, yet its availability often poses a bottleneck to optimal plant productivity [1, 2]. Although P is abundant in many soils, its availability for plant uptake remains limited [3]. While applying soluble P fertilizers can alleviate deficiency, their overuse may lead to excessive P availability and environmental risks [4, 5] and contribute to P loss through runoff causing surface water eutrophication [6] and increased methane emissions [7]. Therefore, a comprehensive understanding of plant adaptive responses to varying P levels is crucial to determine mechanisms for increasing the efficient use of P while maintaining crop yields and environmental stewardship.

Plants manifest a range of adaptive mechanisms that play a decisive role in determining P use efficiency (PUE) under particular P supply scenarios [8, 9]. PUE reflects how efficiently a plant converts absorbed P into biomass. It is influenced by two components: P uptake efficiency (PUpE), which refers to the plant’s ability to absorb P from the environment, and P utilization efficiency (PUtE), which measures how efficiently the plant uses the absorbed P to produce biomass. PUpE is dependent on the ability of roots to acquire available P, which defines P Acquisition Efficiency (PAE), through modifications of morpho-physiological and biochemical properties of roots [10, 11].

Under P deficiency, root growth is enhanced in relation to shoot growth, resulting in an elevated root-to-shoot ratio [11]. In this situation, roots serve an important role as a primary source organ for the delivery of absorbed P to different plant parts while also acting as a strong sink organ for P and carbohydrate to signal meristem activity and root growth [12]. Additionally, to enhance P absorption under P deficiency, roots evolve other adaptive mechanisms such as increasing acid phosphatase (ACP) activity for excreting organic acid anions to the rhizosphere to improve P availability [13] and increasing growth of lateral root and root hairs through production of phytohormone such as indole-3-acetic acid (IAA) and abscisic acid (ABA) [14–16], which can also be stimulated by the colonization of roots by plant growth-promoting rhizobacteria (PGPR) [17]. However, plant-PGPR symbiosis demands significant energy and resources from plants [18, 19], with its efficacy varying depending on the cultivars and the severity of P deficiency [20]. At the molecular level, several P transporter genes are modulated to increase P absorption by roots. Liu et al. [21] documented enhanced expression of the *PHT1* and *PHT2* gene families in potato roots and leaves in response to P shortages. This finding underscores the importance of these two gene families in enhancing P uptake by roots, translocation within shoots, and internal P recycling under deficiency conditions. Although P toxicity is relatively uncommon, it can occur when P is increasingly supplied to P-starved plants that have low capacity to down-regulate their P uptake and avoid elevated P accumulation in leaves [4, 5, 22]. Given the intra-specific difference of plants in response to low and high P supply [22–24], cultivars with higher P efficiency could be an alternate strategy for overcoming the dilemma of deficient and excessive P.

Potatoes play a central role in global food security due to their nutritional properties [25]. Nevertheless, potato productivity has a high P demand caused by a combination of high-yield formation [26] and low PUE, in comparison to other crops, which is attributed to their relatively shallow and less extensive root systems for scavenging P from deeper soil layers [27, 28]. A recent investigation revealed a decline of over 40% in plant biomass and a decrease of more than 20% in sugar concentration in roots of potatoes when exposed to both extremely low (0 mg P L^− 1^) and high P levels (≥ 30 mg P L^− 1^) [11]. This study reaffirmed earlier observations regarding the sensitivity of potatoes to restricted P supply [23, 28, 29] and highlighted the susceptibility of potatoes to conditions with very high P levels. Notably, the response of potatoes to various P conditions varies among cultivars [24]. Moreover, a recent study underscored the efficacy of co-inoculation of potato with plant growth-promoting rhizobacteria (PGPR) in stimulating root growth, thereby enhancing P uptake and plant biomass in potatoes, particularly under P deficiency [11]. Although different PGPR strains can promote plant growth in various ways [30], it is crucial to understand the specific effects of individual strains. Hence, there is a need for further exploration of the mechanisms associated with the alteration of growth as well as P and sugar translocation across different parts of potatoes, particularly under P deficiency.

In this study, we elucidated the responses of two potato cultivars – a table potato (cv. Milva) and a starch potato (cv. Lady Claire) – to varying P levels (0.5–30 mg L^− 1^) in a nutrient solution. Plant growth-promoting *Bacillus subtilis* (*B. subtilis*) was primarily inoculated at low P levels (0.5 and 2 mg L^− 1^) and compared with non-inoculated plants. The selection of this strain was based on its significant presence within the root-associated bacterial community under low P levels, as revealed in our previous investigation [11]. The choice of nutrient solution as the medium for plant growth and PGPR inoculation was to minimize P complexation and diverse microorganisms that occur in the soil and could potentially mask the effects of P and PGPR applications [11]. This study aimed to (I) characterize traits associated with plant morphology, PUE, and molecular adaptations of potato cultivars under varying P levels and (II) evaluate the impact of *B. subtilis* on root system modifications and plant P status. It is hypothesised that the potato cultivars under study will differ significantly from each other in terms of their P efficiency and their reactions to *B. subtilis*. These findings provide the first-hand information that opens new opportunities to further studies on the detailed characterization of these two distinct cultivars, which could be utilized to increase PUE in potato production and guide breeding ventures to develop P-efficient potato cultivars.

## Materials and methods

### Plant materials

Potato cultivars Milva and Lady Claire were sourced from Europlant Pflanzenzucht GmbH, Germany, and C. Meijer B.V., Netherlands, respectively. These cultivars were selected based on their varied responses to P availability, as observed in our previous study [24]. A strain of *B. subtilis* DSM 21393 was obtained from the German Collection of Microorganisms and Cell Cultures (DSMZ, Braunschweig, Germany).

### Experimental setup, plant cultivation, and plant growth-promoting rhizobacteria inoculation

The experiment was conducted using a randomized complete block design with five replications under greenhouse conditions. Supplementary light, emitting a photon flux density of 400 µmol m^− 2^ s^− 1^, was provided on a 16-hour day/8-hour night schedule. The average ambient temperature was maintained at 21.0 ± 1.5 °C. Seed potatoes were germinated for four weeks in nutrient-rich quartz sand, following the protocol outlined in Chea et al. [11]. Subsequently, the seedlings were rinsed with distilled water before being transferred to 6-L pots (one plant per pot) containing a nutrient solution with varying P levels, adjusted with KH_2_PO_4_ and included P levels of 0.5, 2, 5, and 30 mg P L^− 1^ (equivalent to 0.016, 0.065, 0.16, and 0.97 mM P, respectively), labelled as P0.5, P2, P5, and P30, respectively. These P rates cover a wide gradient from P deficiency to potential P toxicity. The selection of these P rates was based on our previous studies by identifying an optimal P supply for potato growth at 5 mg P L^− 1^ in hydroponic solutions, equivalent to 0.2 g P kg^− 1^ in soil [11, 24]. All other nutrients were added at optimal concentrations as described in Additional file 1. The K concentration in each pot was balanced using K_2_SO_4_, and the pH of the nutrient solution ranged between 5.5 and 6.5. The experiment started with an initial nutrient supply of 25% full concentration, which increased to 100% over a week. The nutrient solution was refreshed weekly, with distilled water added between renewals to maintain the volume. Aeration was ensured through polyethylene tubes submerged in the nutrient solution of each pot.

To evaluate the growth-enhancing effects of *B. subtilis*, both P0.5 and P2 treatments received either *B. subtilis* inoculation or no inoculation. For preinoculation, the bacterial strain was cultivated in nutrient broth following the methodology detailed in Chea et al. [11]. A 50 mL aliquot of the *B. subtilis* culture (~ 2.8 × 10^9^ colony-forming units mL^− 1^) was centrifuged at 2,660*g* for 15 min, and the supernatant was discarded. The bacterial cells were suspended in the plant nutrient solution eight days after transplantation (DAT). From this point, bacterial cells were inoculated weekly following the renewal of nutrient solution.

### Plant harvesting and sample preparation

At 25 DAT, a young, fully mature leaf (fourth position from the top) was sampled to assess the nutritional status of the leaves. At 42 DAT, entire plants were harvested and separated into young leaves (fourth from the top), old leaves (bottommost), main stem, residual shoots (including side-shoots and stolons), and roots. Sub-samples from each plant part were ground using liquid nitrogen and stored at − 20 °C. The remaining samples of each plant part were freeze-dried using an EPSILON 2–40 freeze dryer (Christ, Osterode am Harz, Germany) and ground using a hammer mill (DFH 48 Culatti, Kinematica, Malters, Switzerland) with a 0.5 mm sieve.

### Phosphorus and sugar analyses in plant tissue

The P concentration in young leaves, old leaves, stem, remaining shoots, and roots was determined using the method outlined by Koch et al. [31]. The P content for each plant part was determined by multiplying its respective P concentration by the corresponding dry matter (DM). The total P uptake was obtained by summing the P content in both the shoots and roots. The calculation of PAE, PUpE, PUtE, and PUE followed the methodologies outlined by Wacker-Fester et al. [28], Chene et al. [32], and Sandaña [33]:

$$\:\text{P}\text{AE}\text{ = }\frac{\text{Total P uptake (mg }{\text{plant}}^{\text{-1}}\text{)}}{\text{Root DM (mg }{\text{plant}}^{\text{-1}}\text{)}}$$

$$\:\text{PUpE}\text{ = }\frac{\text{Total P uptake (mg }{\text{plant}}^{\text{-1}}\text{)}}{\text{Total applied P (mg }{\text{pot}}^{\text{-1}}\text{)}}$$

$$\:\text{PUtE}\text{ = }\frac{\text{Shoot DM (g }{\text{plant}}^{\text{-1}}\text{)}}{\text{Total P uptake (mg }{\text{plant}}^{\text{-1}}\text{)}}$$

$$\text { PUE }=\text { PUpE } \times \text { PUtE }$$

The soluble sugar concentration within young leaves, old leaves, stem, and roots was determined according to the procedures detailed in Chea et al. [24].

### Root scanning and surface fluorescent labelling

Frozen root samples were defrosted and scanned using an EPSON Perfection V800 Photo scanner (Epson, München, Germany), following the method outlined in Chea et al. [11]. The digital images were analyzed using WinRHIZO image analysis software (Regent Instruments, Québec city, QC, Canada) to determine root length and root surface area. To evaluate the presence of root-associated *B. subtilis*, roots from both inoculated and non-inoculated plants were labelled using Cellbrite Fix Membrane Stains (Biotum, Hayward, USA) according to the manufacturer’s guidelines. The roots were first rinsed with phosphate-buffered saline, then submerged in a 10-fold diluted solution of Cellbrite Fix Membrane Dye and allowed to incubate for 15 min at room temperature. Subsequently, the labelled roots underwent two additional rinses with phosphate-buffered saline before being mounted on a glass slide for imaging with an LSM 780 confocal laser-scanning microscope (Zeiss, Oberkochen, Germany). The Cellbrite Fix Membrane Dye was activated using an argon laser at 488 nm, and emitted light was filtered through a bandpass filter ranging from 493 nm to 630 nm. Image processing was performed using ZEN 2013 software (Zeiss, Oberkochen, Germany).

### Determination of acid phosphatase activity in roots

The ACP activity was determined following the protocols outlined by McLachlan [34] and Bessey et al. [35]. Each root sample (150 mg) was subjected to extraction by adding 1.5 mL of pre-chilled 0.1 M acetic acid buffer, followed by a 1-hour incubation at 4 °C. After centrifugation at 10,000*g* for 25 min, 500 µL of the supernatant was collected and combined with 500 µL of 0.1 M acetic acid buffer and 500 µL of 14.8 mM para-nitrophenyl phosphate solution. This mixture was then incubated at 30 °C for 10 min. To halt the reaction, 500 µL of a 0.5 M NaOH solution was added promptly. The absorbance of the sample was measured at 405 nm using a plate reader (Synergy HTX, Biotek, Winooski, USA). ACP activity was determined by referencing a para-nitrophenol standard using the following equation:

$$\eqalign{& {\rm{ACP\ activity}}\left(\rm{nmol\ min^{{\rm{ - 1}}}{\rm{\ m}}{{\rm{g}}^{{\rm{ - 1}}}}} \right){\rm{ = }}\  ({{{\rm{Absorbance\ at\ 405\ nm - 0}}.{\rm{063}}} \over {{\rm{9}}.{\rm{19}}}}) {\rm{/}}[{\rm{10\ x\ sample\ weight}}({\rm{mg}})] \cr}$$

### Determination of gene expression of phosphorus transporters in *Solanum tuberosum* and the uncharacterized transporter YwkB from *Bacillus subtilis*

Young leaf and root samples collected at 42 DAT were ground using liquid nitrogen. Approximately 100 mg of plant material was used for RNA extraction following the manufacturer’s instructions with the innuPREP Plant RNA Kit (Analytik Jena, Jena, Germany). Subsequently, 750 ng of the total RNA was employed for cDNA synthesis using the iScript™ cDNA Synthesis Kit (Bio-Rad, Hercules, USA), following the manufacturer’s recommendations. Quantitative Polymerase Chain Reaction (qPCR) was performed using the SsoAdvanced Universal SYBR Green Supermix (Bio-Rad, Hercules USA), as per the manufacturer’s instructions and following the protocol described by Koch et al. [31]. Primers for the P transporter genes (*StPHT1;1*, *StPHT1;7*, and *StPHT2;1*) and the housekeeping gene (*StUBIQUITIN*) were sourced from studies by Liu et al. [21] and Koch et al. [31]. Additionally, the expression of the *BsYwkB* gene, identified as a potential metabolite transporter or auxin efflux carrier for *B. subtilis*, was examined via qPCR based on the work of Saier et al. [36]. The forward primer for *BsYwkB* (UniProtKB name: YWKB_BACSU) was 5′-GGA GCG AAT GAA GTT GCG AT-3′, and the reverse was 5′-GGC TCA CAA AGA CCA TGC AG-3′.

### Quantification of phytohormones in roots

Phytohormones were extracted from the frozen ground roots, following the slightly modified protocol of Müller and Munné-Bosch [37]. About 2 g of each sample was suspended in 5 mL of a cold extraction solution (methanol: isopropanol, 20:80) containing 0.1% formic acid [v/v]. This was sonicated at 5–8 °C for 10 min. The suspension was then shaken at 4–5 °C and 390 rpm for 2 h, followed by centrifugation at 15,800*g* for 10 min. An aliquot of 1 mL from each sample was moved to a High Performance Liquid Chromatography (HPLC) amber glass vial and promptly analysed for the concentration of *trans*-zeatin, IAA, ABA, and jasmonic acid (JA).

The assessment was conducted using the HPLC system 1290 Infinity II (Agilent Technologies, Waldbronn, Germany) coupled with the Agilent 6460 triple quadrupole detector (Agilent Technologies, Waldbronn, Germany). Chromatographic separation was performed with a Zorbax Eclipse Plus C18 column, 50 × 2.1 mm, featuring a 1.8 μm particle size (Agilent Technologies, Waldbronn, Germany). The column temperature was maintained at 40 °C, and the injection volume was set at 5 µL. Solvent A comprised water with 0.1% formic acid [v/v], while solvent B was methanol with 0.1% formic acid [v/v]. The gradient was structured as follows: from 0 to 0.2 min, 5% B; from 0.2 to 6 min, an increase from 5 to 75% B; from 6 to 6.50 min, an elevation from 75 to 98% B; from 6.50 to 8.50 min, 98% B; from 8.50 to 9 min, a reduction from 98 to 5% B; and from 9 to 12 min, 5% B. The eluent was ionised via an electrospray ionisation source under the specified parameters. Phytohormones were observed in the multiple reaction monitoring mode. Acquisition parameters are elucidated in Additional file 2. The calibration curve incorporated 12 concentrations, ranging from 0.48 to 1,000 µg L^− 1^. Blank samples were analysed after every seventh sample, while a quality control standard (250 µg L^− 1^) was examined after every 15th sample. The limits of detection (LOD) and limits of quantification (LOQ) were deduced from the standard deviation of the blank [38]. The overall process efficiency was estimated as described by Matuszewski et al. [39] employing the equation:

$$\mathrm{PE}(\%)=\mathrm{C} / \mathrm{A} \times 100$$

C: Peak areas for the isotopically labelled standards spiked before extraction.

A: Peak areas for isotopically labelled standards in pure solvents.

### Statistical analysis

The data obtained from measurements underwent analysis to assess the effects of variable P levels and the inoculation of *B. subtilis* under low P conditions. Analysis of variance (ANOVA) was conducted to determine the significant influences of P levels, cultivars, and their interactions. Furthermore, these interactions were evaluated to compare P treatments within each cultivar and to make comparisons between cultivars at identical P levels. Pairwise comparisons were performed using Tukey’s Honestly Significant Difference (HSD) test at a significance level of *p* < 0.05. The impact of inoculation with *B. subtilis* was assessed by comparing plants with and without the inoculant using a paired t-test at *p* < 0.05 for each P treatment and cultivar. Correlations among the observed traits were determined using Pearson correlation analysis. These statistical analyses followed the methodologies proposed by Gomez and Gomez [40] and were conducted using Statistix 8.0 software (Analytical Software, Tallahassee, USA). Graphical representations were generated using SigmaPlot 12.5 (Systat Software, San Jose, USA).

## Results

### Effect of phosphorus on biomass partitioning and phosphorus status in plants

**Fig. 1:**
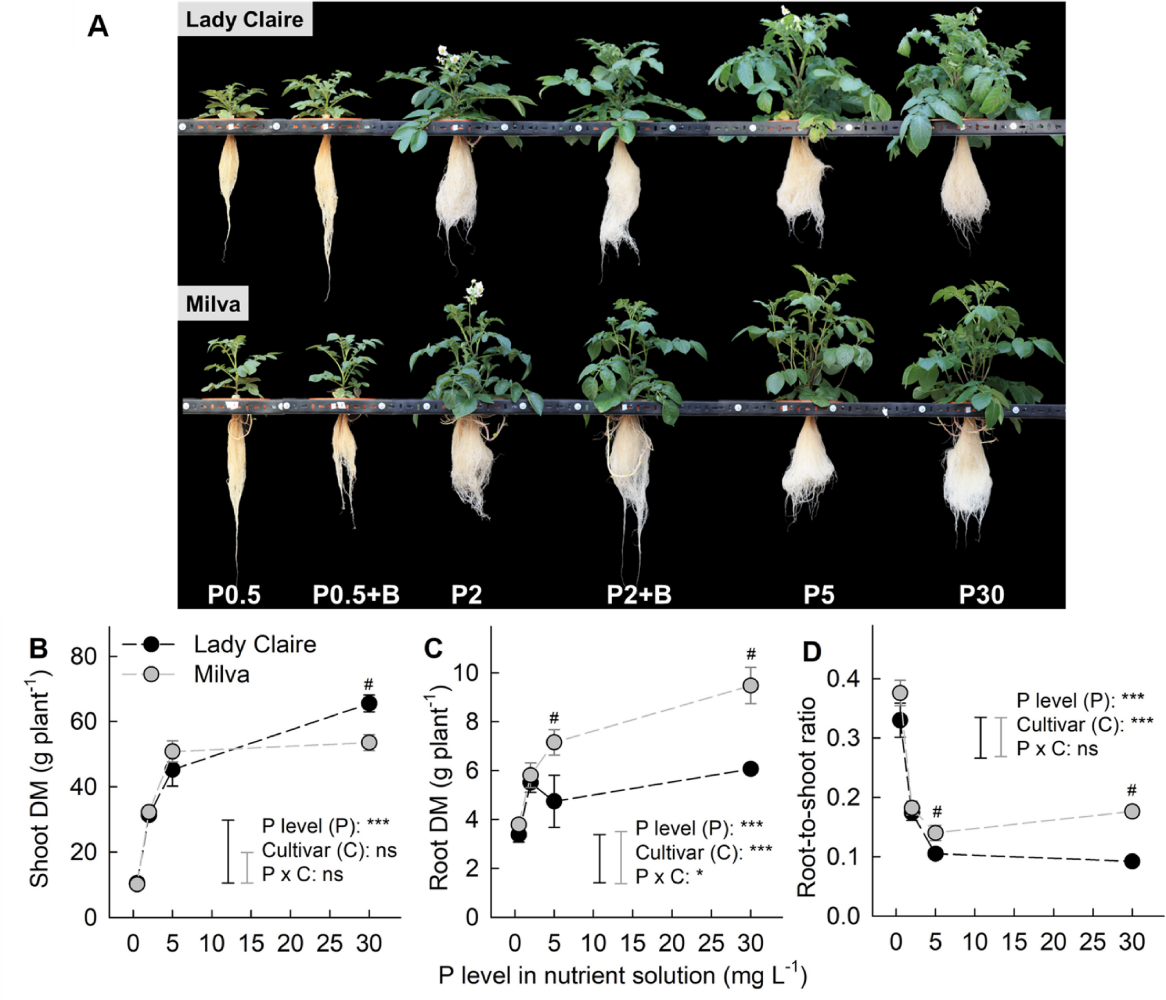
Effects of P availability and *B. subtilis* on plant growth. (**A**) Plant phenotypes and biomass of the potato cultivars Lady Claire and Milva as affected by P availability and *B. subtilis* inoculation, showing the effect of varying P levels (0.5, 2, 5, and 30 mg L^− 1^) on the (**B**) shoot DM, (**C**) root DM, and (**D**) root-to-shoot ratio of the cultivars. Values represent the mean ± SE (*n* = 5). ns, *, and *** indicate non-significant and significant differences at *p* < 0.05 and *p* < 0.001, respectively. Black and grey vertical bars represent critical values for comparisons among P treatments of each cultivar by Tukey’s HSD test at *p* < 0.05. ^#^ indicates a significant difference between cultivars at the same P level, while no indication means non-significant difference. B = *B. subtilis*, DM = dry matter

Phosphorus application exerted a significant influence on the distribution of plant DM and biomass to varying extents (Fig. 1A–D). Both shoot and root DM of Lady Claire and Milva increased with the increasing P level. However, it is noteworthy that the shoot DM of Milva did not exhibit a significant increase beyond P5, and root DM of Lady Claire remained constant beyond P2. As P availability increased, the root-to-shoot ratio of both cultivars declined, yet it surged by 26% for Milva at P30 compared to P5. When comparing the cultivars at identical P levels, Lady Claire exhibited higher shoot DM than Milva at P30. Conversely, across all P treatments, Milva displayed superior root DM and an improved root-to-shoot ratio compared to Lady Claire.

Moreover, P concentration across various plant tissues and on different sampling dates was significantly affected by P level, cultivar, and their interaction (Table 1). For both cultivars, there was a decline of 10–30% in P concentration in young leaves from 25 DAT to 42 DAT across all P levels, except for Milva at P30. At the 42 DAT harvest, an increase was observed in P concentration across different plant sections and P content in both shoots and roots with an increment in P level. Interestingly, the P concentration in roots under P0.5 was 25–30% higher than that under P2. Increasing the P level to P30 led to an elevation in root P concentration, reaching 30.18 mg g^− 1^ for Lady Claire and 16.50 mg g^− 1^ for Milva. There was no significant difference in P concentration among the cultivars in all plant sections under P0.5 and P2. However, at P30, Lady Claire exhibited a higher P concentration in old leaves and roots and an increased P content in shoots and roots. Nevertheless, its P concentration in young leaves and stems was lower than that in Milva.

In this study, PAE, PUpE, PUtE, and PUE were significantly affected by P levels (Table 1). Among the treatments, the highest values for PUpE and PUE were achieved at P0.5. As P availability increased, both PUpE and PUE declined, whereas PAE and PUtE reached their peaks at P5. Both PUpE and PUE in Milva surpassed those in Lady Claire under P0.5, P2, and P5. However, these P efficiency measures were lower in Milva at P30.

**Table 1:** P status in different parts of plant and P efficiency of Lady Claire and Milva under different P levels

Cultivar	*P* level (mg L^− 1^)	P concentration (mg^− 1^ DM)					P content (mg plant^− 1^)		PAE (mg P uptake g^− 1^ root DM)	PUpE (mg P uptake mg^− 1^ applied P)	PUtE (g shoot DM mg^− 1^ P uptake)	PUE (g shoot DM mg^− 1^ applied P)
		Young leaf 25 DAT	Young leaf 42 DAT	Old leaf 42 DAT	Stem 42 DAT	Roots 42 DAT	Shoots	Roots				
Lady Claire	0.5	1.46 ± 0.07^c^	1.17 ± 0.05^c^	1.06 ± 0.05^c^	1.05 ± 0.06^b^	2.17 ± 0.09^b^	12.77 ± 1.24^d^	7.28 ± 0.57^b^	2.78 ± 0.22^b^	0.98 ± 0.07^a^	0.63 ± 0.03^c^	0.62 ± 0.06^a^
	2	1.32 ± 0.07^c^	1.19 ± 0.02^c^	1.07 ± 0.04^c^	1.01 ± 0.06^b^	1.74 ± 0.04^b^	32.80 ± 3.04^c^	9.55 ± 0.65^b^	4.56 ± 0.58^b#^	0.52 ± 0.03^b#^	0.77 ± 0.02^b#^	0.40 ± 0.04^bc#^
	5	2.23 ± 0.16^b^	1.95 ± 0.14^b^	2.65 ± 0.23^b#^	1.42 ± 0.15^b^	2.71 ± 0.24^b^	65.54 ± 9.26^b^	8.60 ± 3.58^b^	8.65 ± 1.44^a^	0.51 ± 0.11^b#^	0.88 ± 0.02^a^	0.52 ± 0.08^ab^
	30	8.41 ± 0.23^a#^	5.81 ± 0.20^a^	7.08 ± 0.15^a#^	4.93 ± 0.24^a#^	30.18 ± 0.87^a#^	367.94 ± 9.72^a^	182.85 ± 5.27^a^	3.02 ± 0.12^b^	0.45 ± 0.02^b#^	0.67 ± 0.01^c^	0.30 ± 0.02^c^
Milva	0.5	1.93 ± 0.07^c^	1.70 ± 0.15^c^	1.46 ± 0.08^b^	0.97 ± 0.03^c^	2.19 ± 0.10^b^	14.16 ± 0.65^d^	8.09 ± 0.25^c^	2.75 ± 0.14^b^	1.08 ± 0.03^a^	0.63 ± 0.02^b^	0.69 ± 0.04^a^
	2	2.19 ± 0.04^c^	1.78 ± 0.05^c^	1.09 ± 0.07^b^	0.92 ± 0.02^c^	1.67 ± 0.07^c^	51.81 ± 3.57^c^	9.56 ± 0.50^c^	6.56 ± 0.70^a#^	0.75 ± 0.04^b#^	0.84 ± 0.01^a#^	0.63 ± 0.04^ab#^
	5	2.96 ± 0.16^b^	2.43 ± 0.12^b^	1.39 ± 0.07^b#^	1.53 ± 0.04^b^	2.62 ± 0.06^b^	112.27 ± 2.45^b^	18.76 ± 1.48^b^	7.12 ± 0.45^a^	0.64 ± 0.02^b#^	0.86 ± 0.01^a^	0.55 ± 0.01^b^
	30	6.02 ± 0.29^a#^	6.47 ± 0.15^a^	4.84 ± 0.28^a#^	6.23 ± 0.09^a#^	16.50 ± 0.21^a#^	309.98 ± 9.73^a^	156.75 ± 9.27^a^	3.01 ± 0.13^b^	0.38 ± 0.02^c#^	0.67 ± 0.01^b^	0.25 ± 0.01^c^
P level (P)		***	***	***	***	***	***	***	***	***	***	***
Cultivar (C)		ns	***	***	***	***	ns	ns	ns	**	ns	**
P x C		***	ns	***	***	***	*	ns	*	*	*	**

ns, *, and *** indicate non-significant and significant differences at *p* < 0.05 and *p* < 0.001, respectively, by ANOVA. Mean values ± SE (*n* = 5) with different letters in the same column indicate significant differences between P levels of each cultivar. ^#^ indicates a significant difference between cultivars at the same P level, while no indication means a non-significant difference. Shoot P content is the combination of P content in all shoot parts including young leaves at 25 DAT. PAE = P acquisition efficiency, PUpE = P uptake efficiency, PUtE = P utilization efficiency, and PUE = P use efficiency

### Quantitative PCR determination of phosphorus transporters

**Fig. 2:**
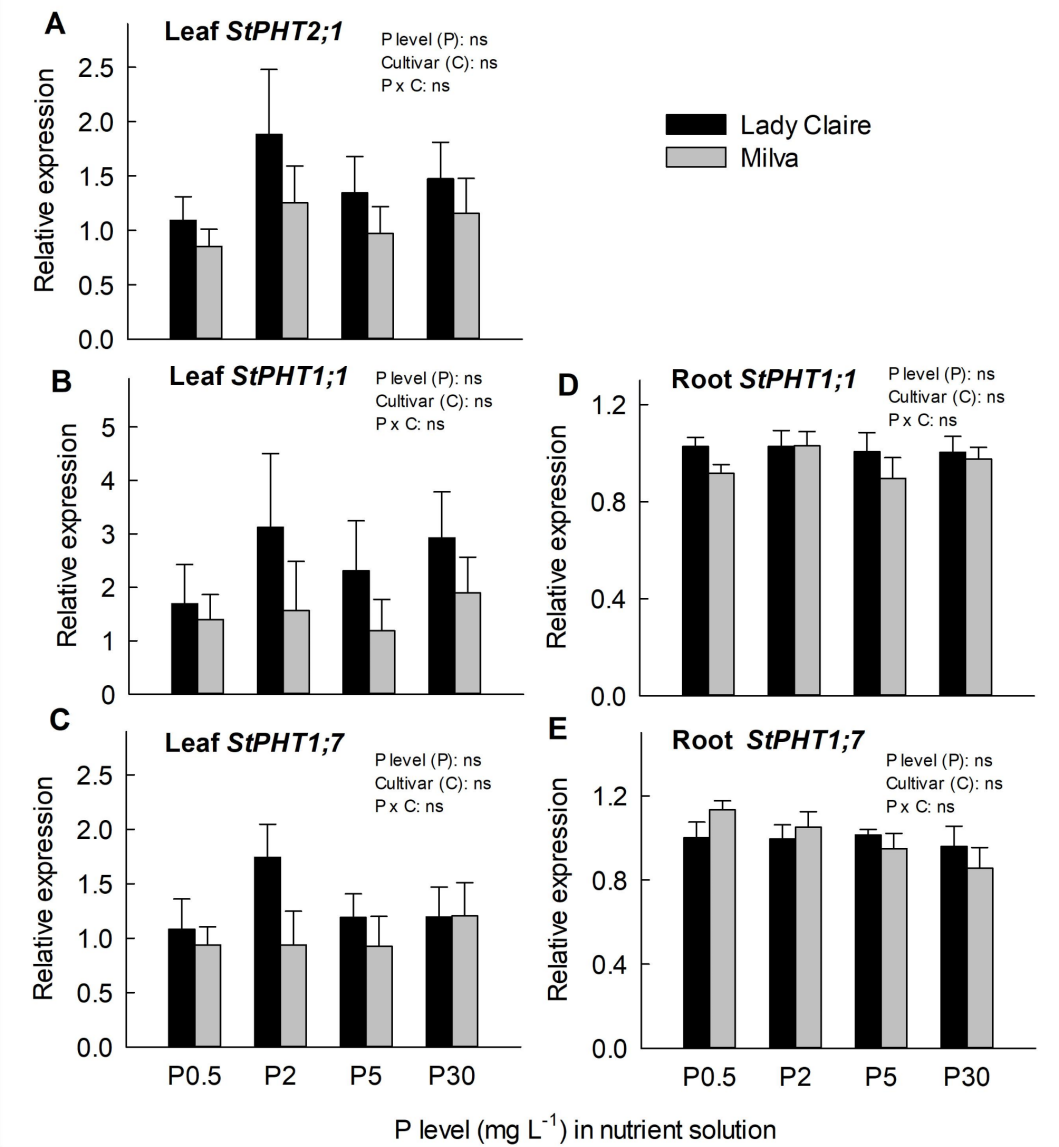
Relative expression of P uptake and translocation genes as influenced by varying P levels. The results indicate the expression level of genes in young leaves and roots at 42 DAT. Transcription in leaves and roots is presented as relative values to a mix of all cDNA from all treatments in leaves and roots, respectively. ns indicates non-significant difference

The relative expression levels of *StPHT1;1* and *StPHT1;7* in both leaves and roots, as well as *StPHT2;1* in leaves were calculated against the expression levels of a mixed cDNA sample consisting of all cDNA samples employed in this study for the respective tissues. When comparing with this common mixed cDNA in leaves or roots, the relative expression levels of *StPHT1;1* and *StPHT1;7* appeared higher in the roots than in the leaves (data not shown). Even though the effects of P level, cultivar, and their interactions were not significant, transcript levels of *StPHT1;1*, *StPHT1;7*, and *StPHT2;1* in leaves seemed elevated in Lady Claire compared to Milva. For roots, there was a notable decline in *StPHT1;7* expression with the rise in P levels (Fig. 2).

### Root morphology, phytohormones, and acid phosphatase activity under varying phosphorus levels

Root DM was notably influenced by both cultivar and P levels. A subset of the fresh root samples were scanned, and relevant parameters associated with the root systems were calculated. Both the total root length and root surface area were significantly impacted by P levels, cultivar, and their interaction (Table 2). The total root length and root surface area were enhanced with increasing P levels up to P2 in Lady Claire and P5 in Milva. Beyond these P levels, no notable increment was observed in both cultivars. Additionally, Lady Claire presented the most substantial proportion of root length and root surface area in fine roots (diameter ≤ 0.5 mm, constituting 98% and 91% of the total root length and root surface area, respectively) at P0.5 relative to elevated P levels. At an identical P level, Milva showcased a more significant total root length and root surface area compared to Lady Claire, predominantly at P0.5, P5, and P30. Nonetheless, the proportion of fine roots in total root length and root surface area was 2–7% higher in Lady Claire under P0.5 compared to Milva. Total root length and root surface area had a significant correlation with total P uptake in Milva, but no such relationships were found in Lady Claire. In both cultivars, specific P uptake and root efficiency displayed a positive correlation with total P uptake (Additional file 3).

**Table 2:** Root morphology and biochemical properties of Lady Claire and Milva under different P levels

Cultivar	P level (mg L^− 1^)	Total root length (10^3^ cm plant^− 1^)	Root length (L) in different diameters (10^3^ cm plant^− 1^)			Total root surface area (10^2^ m^2^ plant^− 1^)	Root surface area (SA) in different diameters (10^2^ cm^2^ plant^− 1^)			IAA (µg g^− 1^)	ACP activity (nmol min^− 1^ mg^− 1^)
			L ≤ 0.5 mm	0.5 < L ≤ 1 mm	L > 1 mm		SA ≤ 0.5 mm	0.5 < SA ≤ 1 mm	SA > 1 mm		
Lady Claire	P0	59.17±5.11^b^	58.17±4.90^b^	0.97±0.22^b^	0.04±0.01^a^	31.12±3.15^b#^	27.52±2.59^b#^	3.44±0.42^b^	0.16±0.06^a#^	0.018±0.008^a^	0.28±0.03^a^
	P2	104.99±5.82^a^	102.01±6.03^a^	2.90±0.20^a^	0.08±0.04^a^	53.04±2.31^a^	50.09±2.78^a^	2.66±0.45^a^	0.29±0.16^a^	0.008±0.001^a^	0.21±0.02^a^
	P5	99.21±2.60^a#^	95.92±2.88^a#^	3.23±0.50^a^	0.06±0.02^a^	53.33±0.85^a#^	48.62±0.83^a#^	4.51±1.02^a^	0.20±0.07^a^	< LOD	0.09±0.02^b^
	P30	90.47±4.82^a#^	87.40±4.50^a#^	3.01±0.41^a^	0.07±0.03^a^	54.14±3.96^a#^	47.78±3.20^a#^	6.12±0.74^a^	0.25±0.11^a#^	0.015±0.000^a^	0.04±0.01^b^
Milva	P0	62.28±4.41^b^	60.41±4.31^b^	1.78±0.12^b^	0.09±0.02^a^	38.56±2.52^c#^	34.77±2.25^c#^	3.44±0.21^b^	0.35±0.08^a#^	0.020±0.005^a^	0.24±0.04^a^
	P2	106.56±9.00^ab^	104.65±8.86^ab^	1.86±0.36^b^	0.05±0.02^a^	58.11±5.66^b^	54.33±5.30^b^	3.57±0.71^b^	0.22±0.09^a^	0.046±0.021^a^	0.23±0.04^a^
	P5	132.47±12.80^b#^	129.51±12.26^b#^	2.90±0.68^ab^	0.07±0.02^a^	77.63±9.51^a#^	71.91±7.68^a#^	5.48±1.29^ab^	0.24±0.09^a^	0.016±0.005^a^	0.14±0.01^b^
	P30	163.10±26.45^b#^	158.91±26.68^b#^	4.03±0.39^a^	0.16±0.07^a^	99.56±9.06^a#^	91.09±9.44^a#^	7.81±0.77^a^	0.66±0.27^a#^	0.021±0.005^a^	0.11±0.05^b^
P levels (P)		***	***	***	ns	***	***	***	ns	ns	***
Cultivar (C)		***	**	ns	ns	***	***	ns	ns	ns	ns
P x C		**	*	*	ns	***	**	*	ns	ns	ns

ns, *, and *** indicate non-significant and significant differences at *p* < 0.05 and *p* < 0.001, respectively, by ANOVA. Mean values ± SE (*n* = 5) with different letters in the same column indicate significant differences between P levels of each cultivar. ^#^ indicates a significant difference between cultivars at the same P level while no indication means a non-significant difference. IAA = indole-3-acetic acid and ACP = acid phosphatase

Phytohormone analysis in roots detected the presence of *trans*-zeatin, IAA, ABA, and JA. However, the concentrations of *trans*-zeatin, ABA, and JA were often below the LOD and LOQ in numerous samples. The influence of P levels, cultivars, and their interaction on root IAA concentration was not considerable. However, the IAA concentration in the roots of Milva was consistently higher than that in Lady Claire across all P treatments (Table 2). In Milva, the peak IAA value was recorded under P2, approximately 2–3 times higher than P0.5, P5, and P30. In contrast, Lady Claire at P2 exhibited the lowest IAA concentration, nearly 50% less than P0.5 and P30. Further analyses determining ACP activity in roots indicated the highest activity at P0.5 in both cultivars, which progressively diminished with increased P levels.

### Effect of phosphorus on sugar concentration in different parts of the plant

Sugar analyses in all plant parts, across every sampling date under different P levels, showed a significant increase in sugar concentration and content in both cultivars with increasing P level (Table 3). In young leaves, the sugar concentration increased from 25 DAT to 45 DAT in every P level for both cultivars, with the sole exception being Milva at P0.5, which saw a 17% reduction in sugar concentration. At 42 DAT, Milva had a diminished sugar concentration in young and old leaves but possessed a heightened sugar concentration in the stem and roots compared to Lady Claire under P0.5, P2, and P5. Conversely, the sugar concentration in young leaves, old leaves, and roots of Lady Claire surpassed that in Milva at P30. Moreover, correlation studies showed generally positive relationships between root sugar concentration and traits like total root length, root surface area, specific P uptake, and root efficiency, though these relationships varied by cultivar (Additional file 4).

**Table 3:** Influence of P supply on sugar partitioning among various parts of plant

Cultivars	P level (mg L^− 1^)	Sugar concentration (mg g^− 1^ DM)					Sugar content (g plant^− 1^)	
		Young leaf 25 DAT	Young leaf 42 DAT	Old leaf 42 DAT	Stem 42 DAT	Roots 42 DAT	Shoots	Roots
Lady Claire	0.5	12.39 ± 0.57^c#^	14.94 ± 1.52^d^	0.74 ± 0.06^b^	22.21 ± 1.79^c#^	11.53 ± 1.10^b#^	0.22 ± 0.04^d^	0.03 ± 0.01^d#^
	2	13.48 ± 1.55^c#^	26.04 ± 4.85^c^	1.54 ± 0.21^ab#^	23.11 ± 2.47^c#^	15.82 ± 1.24^b#^	0.59 ± 0.02^c^	0.08 ± 0.01^c#^
	5	35.72 ± 5.69^b#^	57.20 ± 9.94^b^	1.31 ± 0.13^a^	37.43 ± 4.16^b#^	31.75 ± 3.23^a^	1.81 ± 0.32^b^	0.18 ± 0.01^b#^
	30	55.98 ± 8.53^a#^	84.82 ± 6.78^a#^	1.69 ± 0.10^a^	60.99 ± 1.84^a#^	39.26 ± 3.50^a#^	2.93 ± 0.36^a^	0.24 ± 0.02^a^
Milva	0.5	16.93 ± 1.19^b#^	14.04 ± 0.69^d^	0.80 ± 0.04^b^	29.22 ± 0.96^d#^	14.01 ± 0.84^d#^	0.14 ± 0.01^c^	0.05 ± 0.01^c#^
	2	18.29 ± 0.34^b#^	22.98 ± 1.00^c^	0.92 ± 0.07^ab#^	40.99 ± 1.41^c#^	20.14 ± 1.51^c#^	0.67 ± 0.07^b^	0.12 ± 0.01^b#^
	5	26.94 ± 0.87^a#^	50.04 ± 4.92^b^	1.23 ± 0.13^ab^	72.00 ± 3.45^a#^	35.91 ± 2.36^a^	2.01 ± 0.16^a^	0.26 ± 0.02^a#^
	30	29.69 ± 1.21^a#^	73.28 ± 2.65^a#^	1.39 ± 0.20^a^	68.75 ± 2.29^b#^	26.98 ± 1.03^b#^	2.34 ± 0.23^a^	0.26 ± 0.02^a^
P level (P)		***	***	***	***	***	***	***
Cultivar (C)		*	ns	*	***	*	ns	**
P x C		**	ns	*	***	***	ns	ns

ns, *, * and *** indicate non-significant and significant differences at *p* < 0.05, *p* < 0.01, and *p* < 0.001, respectively, by ANOVA. Mean values ± SE (*n* = 5) with different letters in the same column indicate a significant difference between P levels of each cultivar. ^#^ indicates a significant difference between cultivars at the same P level while no indication means a non-significant difference

### Effect of *B. subtilis* inoculation on P content, sugar levels, gene expression, and root colonization in potato cultivars under low P conditions

**Fig. 3:**
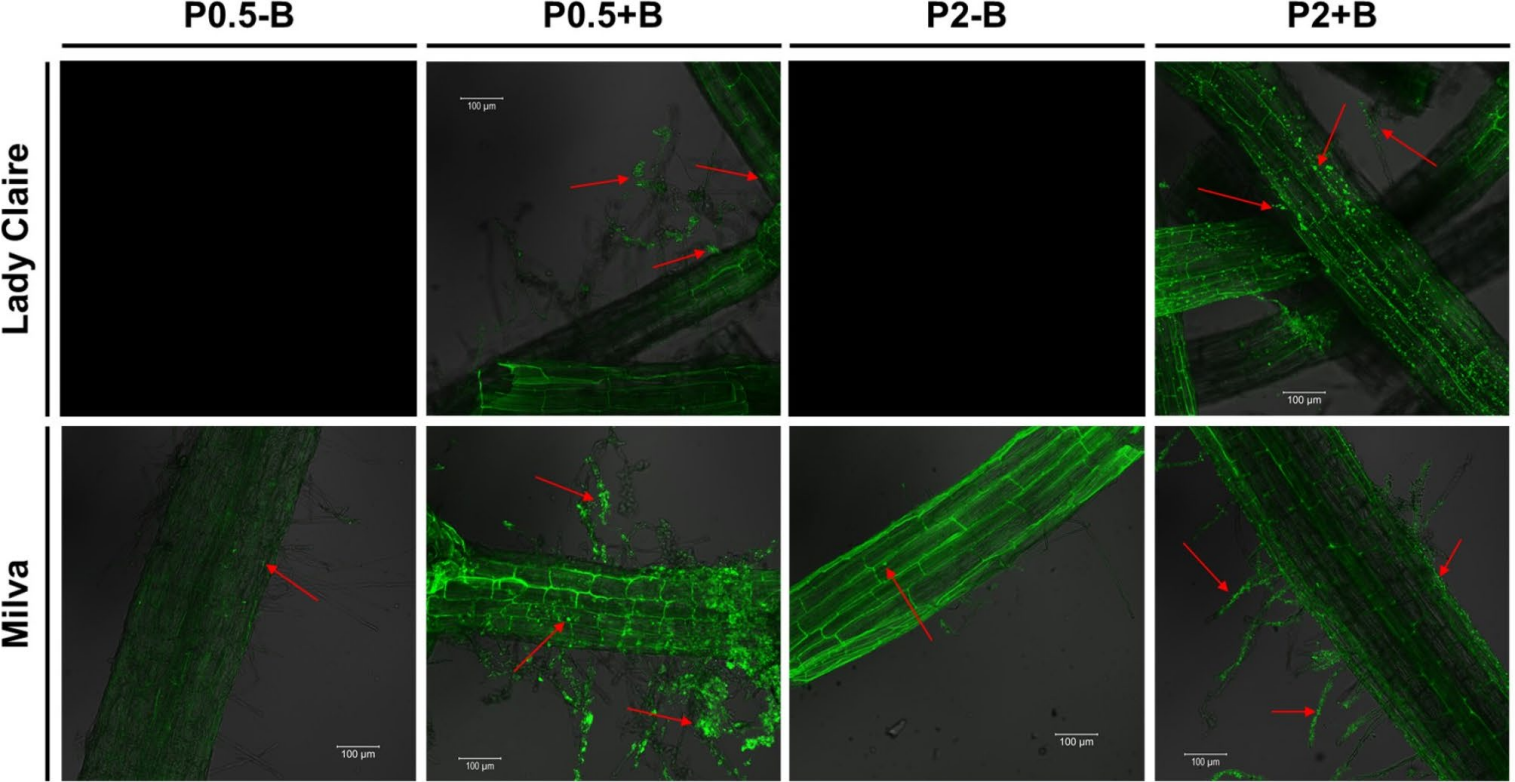
Microscopic images of roots with and without *B. subtilis* inoculation. The images were generated using Cellbrite Fix Membrane Stains. Images for Lady Claire under P0.5-B and P2-B are not available. Arrows indicate the bacterial colonies attached to the roots. B = *B. subtilis*

Fluorescent labeling of root segments with Cellbrite Fix Membrane Stains showed pronounced colonization of root surfaces by *B. subtilis* in inoculated plants compared to non-inoculated controls. (Fig. 3 and Additional file 5). Inoculation with *B. subtilis* significantly increased the relative expression of the *YwkB* gene in roots by 20–110%, depending on the cultivar and P level (Table 4). This suggests attachment and potential internalization of *B. subtilis* in both cultivars.

Additionally, in Lady Claire, *B. subtilis* inoculation increased P content in shoots by 13–25% and in roots by 4–13% at P0.5 and P2 as well as PAE by 10% at P0.5, though this increase was not statistically significant Furthermore, *B. subtilis* inoculation increased the relative expression of *StPHT1;1* and *StPHT2;1* in Lady Claire leaves under P0.5 conditions by 2.2-fold and 1.8-fold, respectively, compared to non-inoculated controls. In Milva, while the effect of *B. subtilis* on P content in shoots and roots was less pronounced, the inoculant still significantly amplified *StPHT1;1* expression by 3.0-fold and *StPHT2;1* expression by 2.15-fold under P0.5 (Table 4).

**Table 4:** Effect of *B. subtilis* on P and sugar content in shoots and roots and gene expression

Cultivar	P level (mg L^− 1^)	*B. subtilis* inoculation	P content (mg plant^− 1^)		PAE (mg P uptake g^− 1^ root DM)	Sugar content in roots (10^2^ mg plant^− 1^)		P transport genes in leaves (relative expression)			*YwkB* gene expression in roots (relative expression)
			Shoots	Roots		Shoot	Roots	*StPHT1;1*	*StPHT1;7*	*StPHT2;1*	
Lady Claire	0.5	-B	12.77 ± 1.24	7.28 ± 0.57	2.78 ± 0.22	2.25 ± 0.36	0.35 ± 0.05^a^	1.69 ± 0.73	1.08 ± 0.28	1.09 ± 0.22	1.38 ± 0.75
		+B	15.86 ± 1.92	8.24 ± 0.76	3.07 ± 0.40	2.95 ± 0.34	0.46 ± 0.05^b^	3.68 ± 1.29	1.52 ± 0.38	1.96 ± 0.38	1.93 ± 0.90
	2	-B	32.80 ± 3.04	9.55 ± 0.65	4.56 ± 0.58	5.89 ± 0.24	0.85 ± 0.10	3.12 ± 1.38	1.74 ± 0.30	1.88 ± 0.60	0.61 ± 0.03^b^
		+B	36.93 ± 1.98	9.87 ± 0.45	4.77 ± 0.25	6.26 ± 0.84	1.03 ± 0.09	2.55 ± 1.17	1.27 ± 0.27	1.39 ± 0.44	1.31 ± 0.01^a^
Milva	0.5	-B	14.16 ± 0.65	7.90 ± 0.28	2.75 ± 0.14	1.41 ± 0.09	0.53 ± 0.09^b^	1.40 ± 0.47^b^	0.94 ± 0.17	0.85 ± 0.16	1.02 ± 0.22
		+B	15.08 ± 0.96	8.25 ± 0.43	2.77 ± 0.16	1.53 ± 0.09	0.70 ± 0.09^a^	4.20 ± 1.08^a^	1.56 ± 0.17	1.83 ± 0.41	1.31 ± 0.15
	2	-B	51.81 ± 3.57	9.56 ± 0.50	6.56 ± 0.70	6.74 ± 0.68	1.15 ± 0.07	1.56 ± 0.92	0.94 ± 0.31	1.25 ± 0.34	1.23 ± 0.85
		+B	51.81 ± 3.15	9.75 ± 0.44	9.34 ± 0.31	7.64 ± 0.88	1.12 ± 0.07	0.57 ± 0.24	0.86 ± 0.17	0.60 ± 0.21	1.42 ± 0.53

Transcription levels are presented as relative values to a mix of cDNA from all treatments. Means ± SE (*n* = 5) followed by different lowercase letters in the same column are significantly different between with and without *B. subtilis* inoculation at the same P level and cultivar. PAE = P acquisition efficiency, B = *B. subtilis*, no indication = not significantly different.

Moreover, *B. subtilis* increased IAA concentration by 65–90% in roots of P0.5 inoculated plants across both cultivars (Additional file 5). The sugar content in the roots of Lady Claire plants inoculated with *B. subtilis* was 20–33% higher than that in non-inoculated plants across both P levels and in Milva at P0.5 (Table 4).

## Discussion

A recent investigation demonstrated that P deficiency and toxicity significantly affected potato growth and metabolite profiles by reducing plant height, biomass, and altering nutrient allocation. The study also showed that co-inoculation with PGPR significantly improved root and shoot biomass under P-deficient conditions, highlighting the potential of PGPR to mitigate the effects of P deficiency [11]. Moreover, another study showed a distinction between the potato cultivars Lady Claire and Milva, identifying Lady Claire as P-inefficient and Milva as P-efficient under conditions of P deficiency [24]. Building upon these findings, the present study sheds further light on the root morphology, physiology, and molecular responses governing the P efficiency of Lady Claire and Milva across different P levels and following inoculation with *B. subtilis*.

### Response of plant dry matter, phosphorus uptake, and root morphology to varying phosphorus availability

A higher variation in root DM among cultivars, in comparison to shoot DM, across all P levels (Fig. 1) indicates that root DM might serve as an indicator of the efficiency of potato cultivars under varying P availability. Given the limited root system of potatoes, improved root growth of Milva is crucial for maximizing P uptake under low P levels [23, 41] and serving as a reservoir for excess P, helping to mitigate potential toxicity under high P levels [22].

In addition to modulating DM distribution in response to P availability, the translocation of assimilated P to distinct plant sections could contribute to discrepancies in P efficiency among cultivars [9, 42]. Both cultivars had an elevated P concentration in the roots under P-deficit conditions, which could be attributed to the plant’s adaptive response by retaining P in the root for a survival strategy. Such a response allows the plant to secure essential P for metabolic processes and growth, despite limited external availability. Similar adaptive mechanisms have been observed in other studies, where increased P storage in roots under low-P conditions was critical for maintaining plant growth [43–45]. Although internal P levels remain severely limited under P deficiency, increased allocation to roots can stimulate further root growth and subsequent P uptake [12]. Despite P allocation within the roots, Milva managed to allocate the scarcely available P to younger leaves, promoting shoot growth. The pronounced P concentration in Lady Claire roots might indicate its ability to retain more P without impeding plant growth under abundant P conditions. Consequently, the P content in the shoots and roots of Lady Claire surpassed that in Milva, reflecting Lady Claire’s abilities in P uptake. This implies reduced P loss in the environment when P is abundantly available.

P uptake within the rhizosphere is predominantly controlled by proton-coupled P symporters of the *PHT1* family [46]. The transcripts of *StPHT1;1* and *StPHT1;7* were identified in roots and leaves, implying a role in P uptake in roots and its translocation to shoots. The slight impact of P levels on the transcription levels of P transporter genes highlighted in this research might be attributed to sampling timing. The P concentration in the nutrient solution rapidly decreased within 24 h post-nutrient renewal, particularly at reduced P levels (data not shown). However, leaf and root samples were taken 42 days post-P treatment initiation and 7 days subsequent to nutrient renewal. The slightly reduced expression of *StPHT1;7* in Milva roots under P30 aligns with the P concentration findings, suggesting limited P uptake activity to avoid toxic P situations in roots. In this study, gene expression was analyzed in young leaves, as they play a critical role in P remobilization during senescence. This is important for understanding nutrient translocation processes under P-limited conditions. Despite this, P translocation to young leaves still occurred, as indicated by the relatively stable expression of *StPHT1;1* (Fig. 2B). Ayadi et al. [48] similarly demonstrated the significance of *AtPHT1;1* in phosphate translocation from roots to leaves under high P conditions in *Arabidopsis*. Transcript levels of *StPHT2;1* and *StPHT1;1* in Lady Claire leaves typically exceeded those in Milva (Fig. 2A, B), suggesting the vital role of these genes in P translocation to young leaves of Lady Claire.

Under low P levels, Milva exhibited higher root DM and P uptake, resulting in enhanced PUpE and PUE compared to Lady Claire. As potato tubers were not accessible under hydroponic conditions at the early harvest (42 DAT), PUE and PUtE determinations were based on the volume of shoot DM produced per unit of supplied P and absorbed P, respectively. The elevated PUtE observed in both cultivars under P2 and P5 revealed its importance in enhancing PUE under both sub-optimal and optimal P conditions, confirming the findings of Wacker-Fester et al. [28] and Sandaña [33]. Dissanayaka et al. [49] demonstrated that PUtE is compromised under P deficiency, potentially due to inefficient energy metabolism for P translocation to shoots, leading to low shoot DM. In environments with high P supply, plants often assimilate more P than they require – particularly in nutrient solutions – until root P uptake diminishes. Surplus P is sequestered to vacuoles to prevent P toxicity in the cytoplasm and simultaneously act as a P reservoir in plants [22, 50]. Consequently, the efficiency of utilising absorbed P in shoot biomass production is reduced. Nevertheless, the PUtE of Milva in this study considerably exceeded that of Lady Claire under less pronounced P deficiency (P2), highlighting the efficiency of Milva in P uptake and utilization at low P levels. Although Lady Claire demonstrated remarkable root P uptake capability and high shoot DM with an abundant P supply, its PUtE was comparable to that of Milva, attributed to the significant volume of absorbed P stored in the roots.

Enhancements in plant PUpE are shaped by alterations in root morphology [10, 23]. Under limited P supply, both the total root length and root surface area of Milva surpassed those of Lady Claire, leading to improved P uptake (Table 2, Additional file 3). Mori et al. [51] also confirmed that an expansive root surface area is important for improving P uptake in conditions of restricted P availability. For Lady Claire, improvement in total root length and root surface area potentially increases P uptake solely under low P level (Additional file 3), given its adaptation to develop finer roots (diameter ≤ 0.5 mm). Conversely, in high P environments, Lady Claire seems to adopt an alternative strategy by sustaining root elongation. These outcomes highlight the efficiency of both Milva and Lady Claire in P uptake under low and high P levels, respectively.

### Responses of root biochemical properties and sugar concentration to varying phosphorus availability

Changes in root morphology, particularly under P deficiency, may be influenced by phytohormones, as documented in barley [15] and tomato [52]. In this study, while an array of phytohormones (IAA, *trans*-zeatin, ABA, and JA) was quantified in the roots, three of them were present at very low concentrations, i.e., below the LOD or LOQ, and were therefore not reported in detail. However, the IAA levels across all P treatments in both cultivars were comparable to those observed in barley roots [15]. Hammond et al. [53] also found that IAA plays a role in lateral root development, root hair elongation, and root density modulation under optimal P conditions. Therefore, the slightly higher IAA concentration in Milva roots compared to Lady Claire might elucidate the role of IAA in enhancing Milva root morphology under both adequate P (P5) and sub-optimal P levels (P2). Furthermore, Rietz et al. [54] suggested that IAA regulates root responses in *Arabidopsis* via its interaction with patatin-related phospholipase. While P levels did not significantly affect IAA concentrations in the roots of either cultivar, Milva exhibited greater variability in IAA levels across different P levels. These findings indicate that Milva roots responded differently to IAA across the various P levels. Our findings provide initial evidence of IAA presence in potato roots, highlighting the need for further research into how IAA influences root morphological changes under different phosphorus conditions. Besides morphological adaptation, both potato cultivars also optimised their use of scarcely available P in roots by amplifying ACP activity at P0.5. Intracellular ACP, an important enzyme in root cells, is crucial for mobilising phosphate and recycling organic P in the vacuole [13].

The primary function of roots is to absorb P and distribute it to other parts of the plant. However, besides the adjustments of phytohormones highlighted above, roots also require significant amounts of P and photoassimilates to alter their morphology, especially when facing P deprivation [12]. P limitation can reduce P concentration in chloroplasts, thereby reducing carbon assimilation [55]. Yet, plants must efficiently translocate available sugars to the roots to maintain root growth and modify the root system while coping with P limitation [56, 57]. In alignment with this, Lemoine et al. [55] and Hermans et al. [58] noted an increased sucrose translocation to the phloem under P deficiency, ready for extended transport to the roots. In this study, sugar concentrations in young leaves of Milva under low P were greater than those in Lady Claire during the early growth phase (25 DAT) but lessened in the later stage (42 DAT). Concurrently, under such conditions, Milva displayed relative sugar accumulation in the stem and roots (Table 3). This may indicate increased phloem loading in Milva, facilitating sugar transport from photosynthetically active tissue to the roots. Conversely, Lady Claire may experience limited sugar translocation to roots under P deficiency, leading to a relatively high sugar concentration in young leaves and sugar content in shoots. However, a high sugar concentration in Lady Claire roots was evident under abundant P, potentially stemming from enhanced specific P absorption and root efficiency. Additionally, the overall positive correlations between root sugar concentration and root traits (Additional file 4) infer that sugar allocation – alongside P – to roots is important for modifying root morphology and P absorption capabilities in response to both low and high P availability in potatoes. While Wissuwa et al. [12] reported that sugar translocation from leaves to roots is not constrained under P deficiency, the extra sugar partitioning in roots by P-efficient cultivars determines root growth and ultimately enhances total P absorption.

### Varitation in the impact of plant growth-promoting *Bacillus subtilis* on rooth growth enhancement under phosphorus deficiency based on cultivar differences

Inoculation with *B. subtilis* enhanced plant P content, PUE, and root morphology under P deficiency across both cultivars. Although the viability of *B. subtilis* in the nutrient solution was not determined in this study, we were able to detect the strain both on the surface and within the intercellular spaces of the roots. This finding concurs with Beauregard et al. [59], who observed the attachment of *B. subtilis* to Arabidopsis roots 24 h post-inoculation. Our recent study also highlighted the presence of this strain at the genus level in association with potato roots. Moreover, we observed the growth of bacterial colonies after plating the nutrient solution on strain-specific agar, with the colonies’ appearance being consistent with that of the original strain [11]. Such observations suggest that the inoculum remains viable in the nutrient solution and adheres to potato roots. In a parallel observation, Eckshtain-Levi et al. [60] documented the colonization of Arabidopsis roots by *B. subtilis* in hydroponic settings.

*B. subtilis* treatment resulted in a more pronounced increase in P content in both the shoots and roots as well as PAE of Lady Claire compared to Milva. This suggests that Lady Claire, when in symbiosis with the plant growth-promoting *B. subtilis*, benefits more in terms of P uptake at very low P levels. Moreover, P translocation genes (*StPHT1;1*, *StPHT1;7*, and *StPHT2;1*) were upregulated in the leaves of *B. subtilis*-treated plants compared to non-inoculated plants. Thus, *B. subtilis* positively impacted both total P uptake and its translocation to young leaves. The enhanced P uptake, modulated by *B. subtilis*, led to enhanced PUE, especially at P0.5, potentially due to improved root morphology, encompassing total root length and root surface area (Additional file 6). The observed increase in total root length and surface area after *B. subtilis* inoculation in this study was approximately half of the increase we reported previously with five different PGPR co-inoculations [11]. Such data imply that inoculating with *B. subtilis* as an individual strain effectively promotes root growth. Given that Lady Claire was inefficient at low P levels, the plant greatly benefited from symbiosis to offset P deficiencies. Conversely, Milva was more resilient to P deficiency, meaning the growth of this cultivar was less dependent on the advantages *B. subtilis* might offer in improving root growth and P uptake. In a previous investigation, *B. subtilis* was found to produce a significant amount of IAA in nutrient broth [11]. Building on this finding, our current study reveals that *B. subtilis* also fosters IAA production in roots, which leads to enhanced root morphology. The increase in IAA content in the roots may be attributed to the influence of *B. subtilis* on hormonal pathways, either by stimulating genes linked to auxin production or through the direct excretion of IAA by the bacteria post attachment or even penetration into the roots. Moreover, *B. subtilis* might produce IAA in the nutrient solution, thereby promoting its uptake by plant roots [61]. IAA has the capacity to regulate primary root growth and root hair formation, resulting in the expansion of both total root length and surface area [17]. In maize, a slight addition of IAA to the nutrient solution (100 µmol IAA L^− 1^) led to a 13% increase in root surface area under P deficiency [62]. This indicates the potential of *B. subtilis* to enhance root morphology in situations of low P level in a P-inefficient cultivar. In contrast, the P-efficient cultivar Milva exhibited tolerance to P deficiency, with its growth less influenced by the plant growth-promoting *B. subtilis*.

## Conclusions

The results of this study underscore significant genotypic disparities among the tested potato cultivars concerning their response to P availability and subsequent P efficiency. Milva exhibited PUE under low P levels by producing more biomass, accumulating more P, and storing additional sugars in its roots. This cultivar also demonstrated responsiveness to IAA, potentially contributing to enhanced root morphology across all P treatments. In contrast, Lady Claire showed limited P and sugar allocation to roots, resulting in less efficient root growth under low P levels, despite having a higher proportion of fine roots. Under high P availability, however, Lady Claire showed increased P uptake, which could be beneficial for reducing P losses and improving environmental sustainability. In conditions of P scarcity, Lady Claire responded to *B. subtilis*, augmenting P translocation to shoots, PUpE, PUE, and root morphology more prominently than Milva. *B. subtilis* also enhanced the specific P uptake and root efficiency of Lady Claire under milder P deficiencies. Therefore, deficit and highly abundant P conditions significantly affected potato growth and metabolite profiles, while inoculation with plant growth-promoting *B. subtilis* improved root and shoot biomass under phosphorus-deficient conditions. These insights may offer valuable guidance for future breeding programs aimed at enhancing PUE in potatoes under diverse P conditions. Further research is imperative to elucidate the mechanisms underlying the impact of IAA on root morphology and the molecular processes governing sugar and P translocation to different plant organs under varying P levels.

## Electronic supplementary material

Below is the link to the electronic supplementary material.

Supplementary Material 1

## Data Availability

All data generated or analyzed in this study is included in this published article and its additional files. The datasets used in the present study are available upon request from the corresponding author.

## Abbreviations

ABA: Abscisic Acid
ACP: Acid Phosphatase
*B. subtilis*: *Bacillus subtilis*
DAT: Days after Transplanting
DM: Dry Matter
IAA: Indole-3-acetic acid
P: Phosphorus
PAE: P Acquisition Efficiency
PGPR: Plant Growth-Promoting Rhizobacteria
PUE: Phosphorus Use Efficiency
PUpE: Phosphorus Uptake Efficiency
PUtE: Phosphorus Utilization Efficiency
