# Supplementary material for: Adaptation of potato cultivars to phosphorus variability and enhancement of phosphorus efficiency by Bacillus subtilis
Source: BMC Plant Biol. 2024 Dec 12;24:1176. doi: 10.1186/s12870-024-05868-x (PMC11636052; doi:10.1186/s12870-024-05868-x)
Supplement: Supplementary file 1 — Supplementary Material 1 [file 12870_2024_5868_MOESM1_ESM.pdf]

Additional files

Additional file 1. Concentration of nutrients applied in the hydroponic system

Nutrient	Concentration (mg L^{-1})	Nutrient sources
P	0 - 30	KH_2PO_4
N	56.04	NH_4NO_3, $Ca(NO_3)_2$* $4H_2O$
K	104.25	K_2SO_4
Ca	69.05	$Ca(NO_3)_2$* $4H_2O$, $CaCl_2$* $2H_2O$
S	45.01	K_2SO_4, $MgSO_4$* $7H_2O$, $CuSO_4$* $5H_2O$, $ZnSO_4$* $7H_2O$, $MnSO_4$* H_2O
Mg	1.62	$MgSO_4$* $7H_2O$
Cu	0.02	$CuSO_4$* $5H_2O$
EDTA-Fe	5.58	$C_{10}H_{12}FeN_2NaO_8$* $3H_2O$
Zn	0.09	$ZnSO_4$* $7H_2O$
B	0.14	H_3BO_3
Mo	0.13	$H_{24}Mo_7N_6O_{24}$* $4H_2O$
Mn	0.07	$MnSO_4$* H_2O
Na	2.32	$C_{10}H_{12}FeN_2NaO_8$* $3H_2O$
Cl	2.83	$CaCl_2$* $2H_2O$

Additional file 2. Acquisition parameters for phytohormone analysis.

Compound	RT [min]	Polarity	Parent Ion [m/z]	Fragmentor V	Collision Energy V	Product Ion [m/z]
Trans-zeatin	1.93	+	220.1	100	15	136.1
					9	202.1
					22	148.1
Trans-zeatin-d5	1.92	+	225.1	105	16	137.1
					10	207.2
Indoleacetic acid (IAA)	3.88	+	176.1	84	14	130
					38	103.1
					40	77.2
Indoleacetic acid-d4	3.87	+	180.1	84	15	133.2
					38	106.1
					45	79.2
Abscisic acid (ABA)	4.68	-	263.1	85	4	153.1
					5	219.1
					12	204.1
Abscisic acid-d6	4.67	-	269.1	88	4	159.1
					8	225.1
Jasmonic acid (JA)	5.20	+	211.2	85	8	133.1
					8	151.1
					5	193
Jasmonic acid-d5	5.19	+	216.1	85	8	135.1
					9	153.2
					7	198.2

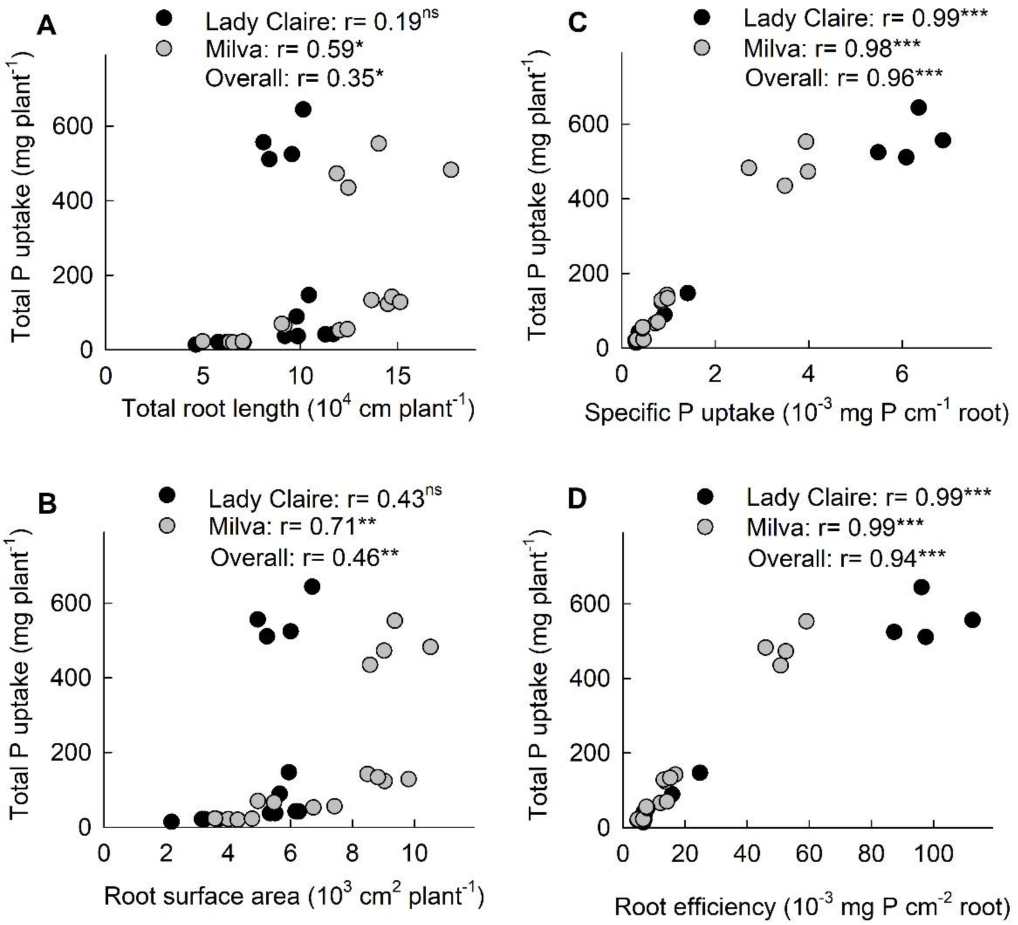

Additional file 3. Correlation between root morphological traits and total P uptake. ns, *, and *** indicate non-significant and significant differences at $p<0.05$ and $p<0.001$, respectively, by Pearson correlation.

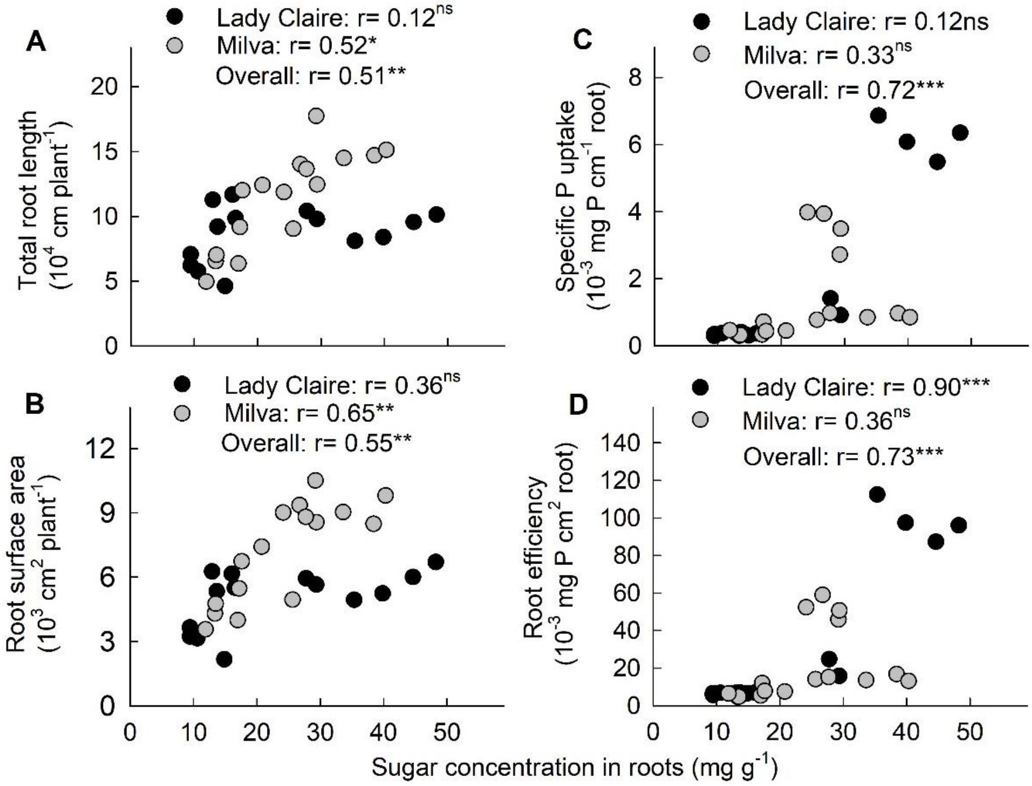

Additional file 4. Correlation between root sugar concentration and root morphological traits. ns, *, and *** indicate non-significant and significant differences at p<0.05 and p<0.001, respectively, by Pearson correlation.

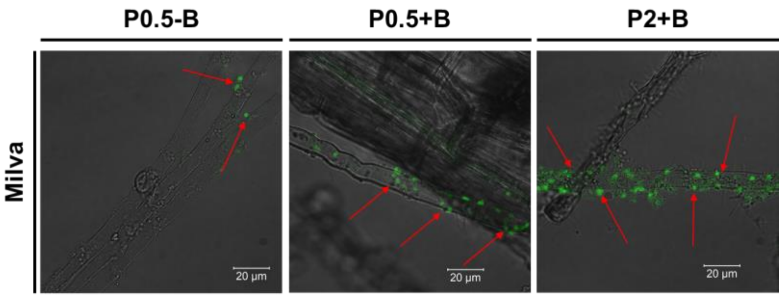

Additional file 5. Microscopic images of roots of cultivar Milva with and without *B. subtilis* inoculation using Cellbrite Fix Membrane Stains. Arrows indicate the bacterial colonies attached to the roots. B = *B. subtilis.*

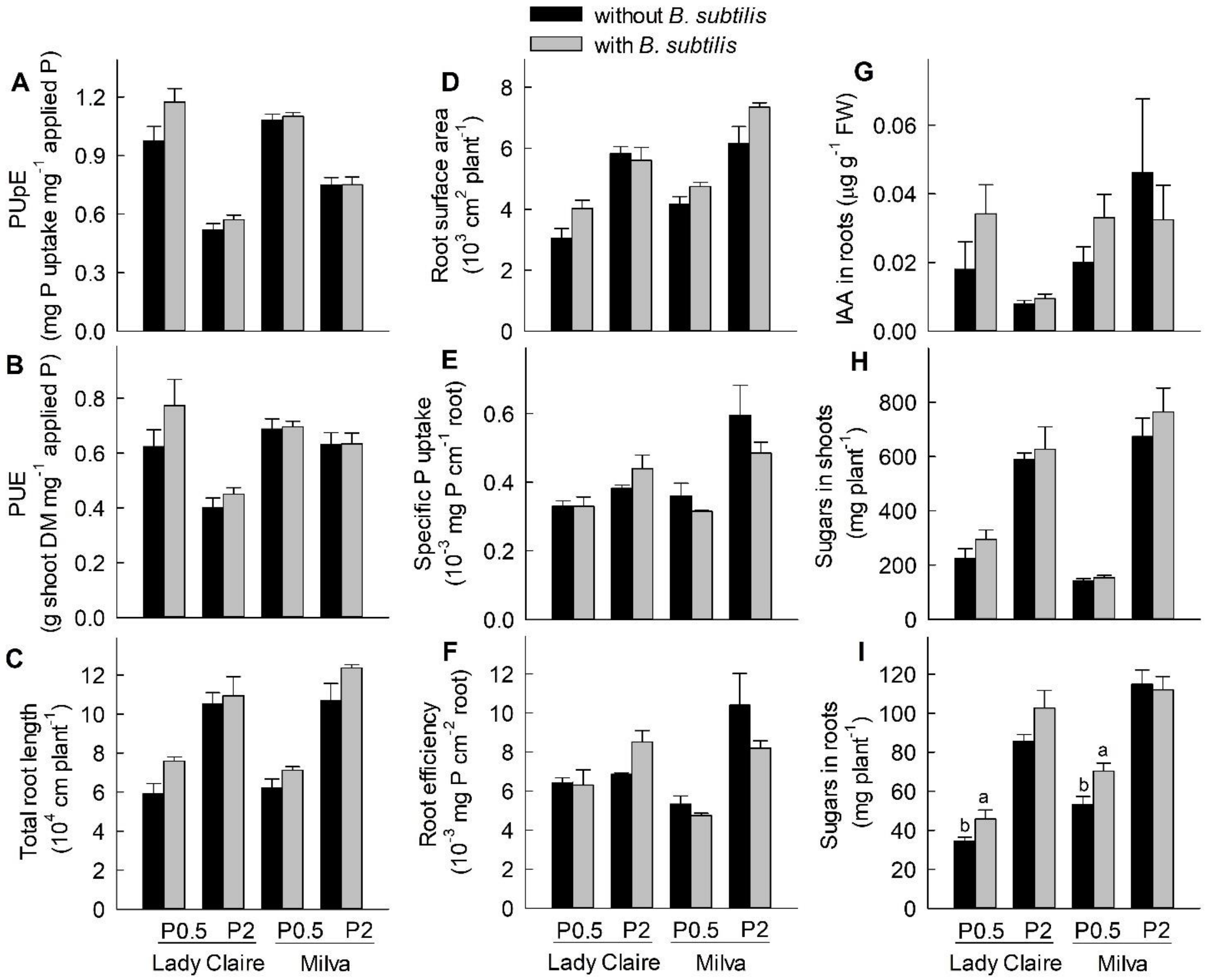

Additional file 6. Effect of *B. subtilis* inoculation on (**A**) PUpE, (**B**) PUE, (**C-F**) root traits, (**G**) root IAA, and (**H-I**) sugar content in shoots and roots. Mean ± SE (n=5) with different lowercase letters are significantly different at the same P level and cultivar. No indication = not significantly different.
